# Real-word data on autologous stem cell transplantation in older patients with multiple myeloma

**DOI:** 10.1007/s00277-019-03878-6

**Published:** 2019-12-10

**Authors:** Frida Schain, Annica Dominicus, Fredrik Borgsten, Marlene Mozart, Magnus Björkholm

**Affiliations:** 1 Schain Research, Grönviksvägen 14, SE-161 76 Bromma, Sweden; 2 Department of Medicine, Division of Hematology, Karolinska University Hospital Solna and Karolinska Institutet, SE-171 76 Stockholm, Sweden; 3 Scandinavian Development Services, SDS, Svärdvägen 23, SE-182 33 Danderyd, Sweden; 4 Janssen-Cilag, Medical Affairs, Kolonnvägen 45, SE-170 67 Solna, Sweden

Dear Editor,

It was with great interest we read the article by Marini et al. regarding outcomes in older patients with multiple myeloma (MM) undergoing autologous stem cell transplantation (ASCT) recently published in the Journal [1]. We and others have described a rapid increase in MM incidence and prevalence in Western countries over the last decades. This is mainly due to an aging general population and more effective treatment options which have resulted in better survival outcomes [2, 3]. Given the introduction of novel treatment alternatives, it is important to define the future role of high-dose treatment and ASCT. Marini and co-authors concluded that age was not associated with event-free survival (EFS) or overall survival (OS). However, the cohort was small (*n*=132) and originated from one centre. We would therefore like to contribute with additional results from a population-based national cohort including all MM patients who underwent ASCT in Sweden between 2005 and 2016 (*n*=1479). Median age at transplantation was 60 (interquartile range [IQR] ^25,75^ 54.0–64.0) years, and 281 patients (19%) were > 65 years at ASCT. Notably, median age of patients undergoing ASCT was lower in the group diagnosed before 2013 versus after 2013, confirming previous reports describing a trend of transplanting older patients [4, 5].

Patients were stratified by age at transplantation (≤ 50, 51–54, 55–59, 60–64, ≥ 65 years) and sex. From initiation of first line treatment, univariate analyses showed a median time to next treatment (TTNT) of 3.0 (95% confidence interval [CI] 2.9–3.2) years, with no differences between age groups (*p* = 0.41) or between males and females (*p* = 0.48). A trend analysis demonstrated that median crude OS from start of induction therapy was 7.5 (95% CI 6.8–8.0) years and there was a statistically significant difference between age groups (*p* < 0.0001), with longer OS for younger patients.

Multivariate Cox regression models showed that high age and ASCT before 2013 were associated with inferior TTNT and OS. Exposure to consolidation treatment was associated with shorter TTNT up to one year after induction treatment initiation, and shorter OS during the first two years after induction treatment initiation; however, these results should be interpreted with caution since we were unable to control for clinical risk factors or tumour status prior to ASCT. Given the fact that Swedish treatment guidelines did not recommend consolidation treatment until 2017 to patients with normal cytogenetic profiles, one may speculate that patients receiving consolidation had a more aggressive disease and consequently responded less well to treatment. Also, the definition of consolidation/maintenance treatment was less stringent compared with most clinical trials (Tables 1 and 2).

**Table 1:** Time-varying multivariate Cox regression models for TTNT adjusted for age, sex, year of diagnosis, type of induction treatment, and exposure to consolidation and maintenance treatment

Variable	Hazard ratio (95% CI)
Age at diagnosis (< 50 years, reference)	
50–64 years	1.22 (1.00–1.48)
≥ 65 years	1.36 (1.07–1.72)
Sex (female, reference)	
Male	1.03 (0.90–1.17)
Year of diagnosis (2005–2012, reference)	
2013–2016	0.72 (0.62–0.84)
Type of induction regimen (IMiD-containing, reference)	
Non-IMiD-containing	1.17 (0.96–1.43)
Exposure to consolidation therapy (no, reference)	
Yes, < 1 year*	8.79 (5.84–13.25)
Yes, 1–2 years*	1.82 (1.08–3.07)
Yes, ≥ 2 years*	1.08 (0.60–1.92)
Exposure to maintenance therapy (no, reference)	
Yes	1.16 (0.85–1.59)

*TTNT*, time to next treatment; *OS*, overall survival; *CI*, confidence interval; *IMiD*, immunomodulatory drug

*Refers to time from induction treatment initiation

**Table 2:** Time-varying multivariate Cox regression models for OS adjusted for age, sex, year of diagnosis, type of induction treatment and exposure to consolidation and maintenance treatment

Variable	Hazard ratio (95% CI)
Age at diagnosis (< 50 years, reference)	
50–64 years	1.62 (1.23–2.13)
≥ 65 years	2.13 (1.55–2.93)
Sex (female, reference)	
Male	0.90 (0.76–1.06)
Year of diagnosis (2005–2012, reference)	
2013–2016	0.74 (0.59–0.93)
Type of induction regimen (IMiD-containing, reference)	
Non-IMiD-containing	1.21 (0.93–1.58)
Exposure to consolidation therapy (no, reference)	
Yes, < 1 year*	9.45 (5.25–17.00)
Yes, 1–2 years*	3.87 (2.11–7.08)
Yes, ≥ 2 years*	1.19 (0.6 5–2.18)
Exposure to maintenance therapy (no, reference)	
Yes	0.80 (0.51–1.24)

*TTNT*, time to next treatment; *OS*, overall survival; *CI*, confidence interval; *IMiD*, immunomodulatory drug

*Refers to time from induction treatment initiation

Survival rates have improved in MM mainly due to modern drugs and ASCT. There has been a trend of increased autografting of older MM patients during the last decade. However, improvement in survival outcomes are less pronounced in older patients and increasing age is still a predictor for worse outcomes. The rapid introduction of new treatment options will hopefully also benefit older patients and may change the future role of high-dose treatment and ASCT.
